# Perioperative Care of Children with Severe Neurological Impairment and Neuromuscular Scoliosis—*A Practical Pathway to Optimize Peri-Operative Health and Guide Decision Making*

**DOI:** 10.3390/jcm11226769

**Published:** 2022-11-16

**Authors:** Giuliana C. Antolovich, Monica S. Cooper, Michael B. Johnson, Kris Lundine, Yi Yang, Katherine Frayman, Moya Vandeleur, Ingrid Sutherland, Donna Peachey, Tali Gadish, Ben Turner, Adrienne Harvey

**Affiliations:** 1 Department of Neurodevelopment & Disability, Royal Children’s Hospital, 50 Flemington Road, Melbourne, VIC 3052, Australia; 2 Neurodisability and Rehabilitation, Clinical Sciences, Murdoch Children’s Research Institute, 50 Flemington Road, Melbourne, VIC 3052, Australia; 3 Department of Paediatrics, University of Melbourne, 50 Flemington Road, Melbourne, VIC 3052, Australia; 4 Department of Orthopaedics, Royal Children’s Hospital, 50 Flemington Road, Melbourne, VIC 3052, Australia; 5 Gait Lab and Orthopaedics, Clinical Sciences, Murdoch Children’s Research Institute, 50 Flemington Road, Melbourne, VIC 3052, Australia; 6 Department of Respiratory and Sleep Medicine, Royal Children’s Hospital, 50 Flemington Road, Melbourne, VIC 3052, Australia; 7 Respiratory Diseases Group, Murdoch Children’s Research Institute, 50 Flemington Road, Melbourne, VIC 3052, Australia; 8 Centre for Health Analytics, Royal Children’s Hospital, 50 Flemington Road, Melbourne, VIC 3052, Australia; 9 Paediatric Intensive Care Unit, Royal Children’s Hospital, 50 Flemington Road, Melbourne, VIC 3052, Australia; 10 Paediatric Intensive Care, Clinical Sciences, Murdoch Children’s Research Institute, 50 Flemington Road, Melbourne, VIC 3052, Australia; 11 Department of Anaesthetics, Royal Children’s Hospital, 50 Flemington Road, Melbourne, VIC 3052, Australia; 12 Anaesthetics, Clinical Sciences, Murdoch Children’s Research Institute, 50 Flemington Road, Melbourne, VIC 3052, Australia

**Keywords:** cerebral palsy, severe neurological impairment, scoliosis, shared decision-making

## Abstract

Neuromuscular scoliosis is a common feature in children with severe neurological impairment (SNI), including those with severe cerebral palsy. Surgical correction of scoliosis is the mainstay of treatment. This group of patients also have associated medical complexity. The complication rates post-surgery are high, although, for many, they are worth the risk. There are currently no published practice guidelines or care pathways for children with SNI who are undergoing scoliosis corrective surgery. In response to the high uptake of this surgery, coupled with the expected complication rates, our hospital established a perioperative clinic. The purpose of this paper is to describe our perioperative approach. This clinic has developed into a service beyond perioperative care and, with the collaborative meeting, enables shared decision-making to identify the right candidate for surgery. The process involves surgical expertise, understanding the family and child at the centre, and optimisation of medical care pre- and post-surgery. In this paper, we describe the process in a step-by-step manner. We provide clinical vignettes, as well as the proformas that we use, and we highlight the benefits of the team-based process.

## 1. Introduction

Neuromuscular scoliosis is very common in children with a physical disability, particularly in those who function at the more severe end of the motor disability spectrum. The group at high risk of neuromuscular scoliosis includes children with Severe Neurological Impairment (SNI) [1], defined as children with diseases of the central nervous system, with permanent motor and cognitive impairment, with both static and progressive disorders [1], and those with cerebral palsy (CP) and who function within Gross Motor Function Classification System (GMFCS) levels IV and V [2,3]. These children also have significant medical co-morbidities [4,5]. It is likely that weaknesses in the postural muscles and diaphragm contribute to neuromuscular scoliosis, which plays a significant role in the evolution of chronic lung disease and respiratory failure in this population [6].

Advances in medical care, such as access to neonatal and paediatric intensive care, management of epilepsy and infection, and technology support (such as non-invasive ventilation and supplemental nutrition), coupled with societal changes and expectations, have substantially modified the survival of children with SNI and increased the number of children living with medical complexity [7]. For many, this increased longevity is associated with acquired morbidity and medical fragility [7]. Severe and untreated neuromuscular scoliosis is an increasingly apparent issue. Over time, the scoliosis may become stiffer, and the consequences of this include difficulties maintaining the head in the midline to continue with adequate socialisation, loss of sitting abilities, pressure sores and reduced pulmonary function [5]. Scoliosis is also a frequent cause of pain [8,9,10,11].

Whilst surgical correction is the mainstay of treatment for neuromuscular scoliosis [5,12], it is not without complications [13]. The population of children who require this surgery are medically complex, and as a consequence, decision-making to ensure optimal outcomes is important. The aim of the surgery is to align the spine and balance the head, shoulders, and trunk over a level pelvis [5]. This, in turn, improves quality of life [5,14,15,16]. Parents/carers have reported this surgery to be “the most beneficial intervention in their child’s life” [15]. Only one study has shown improvements in lung function post-surgery [17], whilst others show pneumonia as a major complication [18]. Although the satisfaction rates postoperatively are very high, complications from surgery remain significant [19]. Overall, high-quality evidence on post-surgical outcomes is still lacking, particularly for outcomes other than curve correction [13].

Decision-making about interventions for children who are complex and medically fragile creates challenges for parents and the clinical team. Deciding when medical and surgical interventions are helping or harming a child in these circumstances is clinically and ethically complex, and there is a substantial obligation to thoughtfully approach decision-making for this group. Balancing the burdens and risks of treatments with benefits for a given child requires a collaborative multidisciplinary view, anticipatory care and active engagement with parents and carers.

This paper will describe the approach to the perioperative care of children with SNI with neuromuscular scoliosis adopted in a tertiary medical centre in Australia. The approach to care has been developed to support complex decision-making for children with SNI [1,20]. An anticipatory approach is needed to ensure that health is optimised prior to any surgical intervention. However, the goals of the surgery and broader goals of care need to be considered when deciding who will be an appropriate surgical candidate. The practical details of our approach and case vignettes are provided. The team approach to care has resulted in broader benefits, which will be described.

## 2. The Care Pathway

A recognition of the increasing medical complexity and frequent postoperative complications of the population requiring surgical management for spinal deformity has been a driver for the development of a clinical care pathway to address the needs of this higher-risk group [13]. A project was established to develop this pathway using the expertise of staff from the Divisions of Medicine, Surgery, Critical Care and Allied Health, and research partners from the Murdoch Children’s Research Institute (MCRI). This project resulted in the establishment of two additional clinical services to support decision-making and perioperative care: the Medical Neuromuscular Scoliosis Clinic (hereafter referred to as Clinic) and the Neuromuscular Scoliosis Multidisciplinary Meeting (hereafter referred to as Meeting).

Ambitious goals for surgical and peri-operative care in surgery for children with neuromuscular scoliosis include decreasing the complication rate to <10%, reducing Intensive Care Unit admission to <24 h and reducing hospital admission lengths to <7 days [21]. Another important consideration is to address whether the institution can manage the level of medical and surgical complexity [21]. Preoperative assessment clinics have been shown to be cost-effective and paediatricians have been shown to make a number of recommendations for medical management [22,23]. The team approach, with detailed perioperative planning and postoperative management, is now considered a mainstay of the treatment for correction of neuromuscular scoliosis [21]. In a recent study, 77% of surgeons reported adhering to preoperative protocols for children with CP within their centres, although there was marked variation in the described peri-operative care [24]. There are established protocols for children with neuromuscular scoliosis undergoing corrective surgery [25]. However, these are developed for children with other conditions and focus on peri-operative care rather than team-based decision-making.

## 3. The Clinical Setting

The Orthopaedic Department provides clinical care for the assessment and management of children with scoliosis. Children are referred to this clinic from multiple sources—from within the hospital, from other major centres in the state (both metropolitan and rural), from community-based clinic services (public and private) and from interstate services. Children with a range of aetiological diagnoses are seen in this clinic. Routine care in these clinical services includes the imaging, assessment, and consideration of non-surgical (expectant care or bracing) and a range of surgical options.

## 4. Medical Neuromuscular Scoliosis Clinic (Clinic)

**Figure 1:**
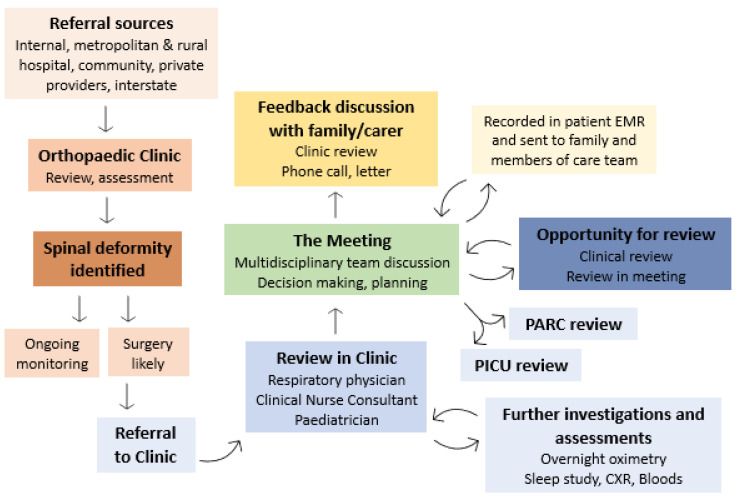
Description of clinical assessment and process of the Clinic and the Meeting. A summary of the outcomes of the clinical case conference, which includes further investigations and details of the peri-operative plan are included in the patient Electronic Medical record (EMR), allowing for access for all members of the team as a reference point for peri-operative management and admission. (PARC—pre-anaesthetic review clinic, PICU—Paediatric Intensive Care Unit, CXR—Chest X-ray).

Children identified in the orthopaedic clinic as potential candidates for surgical intervention are referred to the Clinic (Figure 1). Referrals to this clinic include children with cerebral palsy, SNI, both static and progressive conditions (including Rett syndrome, cerebral palsy-like conditions, neural tube defects, genetic conditions resulting in a motor disability), and other neurodisabilities (Prader Willi Syndrome, intellectual disability syndromes). Children with neuromuscular disorders (e.g., Duchenne, Spinal Muscular Atrophy) are currently assessed in an alternative multidisciplinary setting within the hospital and are not routinely seen in the Clinic, though this process is changing. A review of referrals to the Clinic suggests that almost 40% of children referred to this clinic receive their primary care outside of our hospital.

The Clinic is led by a neurodevelopmental/complex care paediatrician and includes a respiratory physician and a neurodevelopmental clinical nurse consultant. The goals of the Clinic are to (1) identify and assess the medical comorbid conditions and risk factors for each child, (2) take the opportunity to optimise health prior to surgery and, most importantly, (3) support decision-making about proceeding with corrective surgery.

To better understand the potential benefits and risks for each child, the health, well-being and co-morbid conditions are reviewed, and the goals of the surgery as identified by the family (and the child where possible) are defined and clarified. An assessment of the potential risks and identified benefits are incorporated into both decision making and planning of perioperative care.

A detailed medical history, including respiratory history, feeding and nutrition, epilepsy control, movement disorder, sleep, and pain history, is collected. Communication, behavioural and sensory issues, schooling, and supports—both home and community based—are also elicited to better understand the issues that will face the child and family, both as inpatients and as barriers to discharge and recovery (Table S1).

In most cases, the child will have a baseline nutritional blood panel completed, including a capillary acid base following the appointment. Co-morbid conditions that may impact the surgery or recovery are identified and addressed to optimise the preoperative health of the child. Additional investigations—for example, chest radiograph, overnight oximetry, or polysomnography—will also be requested at this time depending on the clinical need. A perioperative care plan, which might include admission for a “tune-up”, a nutritional assessment and optimisation, optimisation of respiratory health, drooling, tone and movement disorder, is prepared (File S1).

An important goal of the Clinic appointment is the exploration with the family, and child where possible, of the goals they have for the surgery, what they hope the surgery will achieve for the child, and their primary concerns or worries about the surgery. Realistic goals include reduction in pain, easier care, improved ability to perform activities of daily living, and improved social interaction [26]. This discussion also involves consideration of the overall goals of care for a child and whether there is an advanced care plan in place. If this is the case, a suspension of the advanced care plan will be required during the peri-operative period, and this must be discussed not only with the family but with the broader team.

## 5. Neuromuscular Scoliosis Multidisciplinary Meeting (Meeting)

The Meeting follows the Clinic and brings together clinicians from different craft groups linked to the service—orthopaedic surgeons, respiratory physicians, orthopaedic clinical nurse consultant, neurodevelopmental nurse consultant, paediatricians, allied health clinicians, research allied health clinician, anaesthetist, and a paediatric intensive-care physician. In some cases, the primary or lead paediatrician of the child and other subspecialists (e.g., respiratory physician from another site, cardiologist), who are part of the child’s care team, are invited to join the discussion.

The clinical history and key clinical factors, including a description of the family’s and child’s goals and concerns, are presented and discussed. This discussion explores and highlights the potential benefits to the child and the identified risks. If the child is a suitable candidate for surgery, a detailed perioperative plan is developed, including identification of any additional investigations or management required to optimise the health of the child (File S1).

An important goal of this discussion is to determine whether a child will benefit from an elective admission to the Paediatric Intensive Care Unit (PICU) to receive their postoperative care. There are some factors that help predict the need for postoperative PICU care: a significant respiratory history and previous admissions to PICU [27,28], an established need for non-invasive ventilation [28], and possibly a higher identified risk in certain diagnostic groups—for example, girls with Rett syndrome [29]. A history of epilepsy or a previous admission to PICU with a respiratory illness both increase the risk and length of stay in PICU post-surgery [28,30]. The surgical plan is a significant factor in this decision (minimally invasive instrumentation versus spinal fusion). Clarity regarding the need for and benefits of a PICU admission is very important. Whilst there are advantages to an elective admission to PICU for postoperative care (one-on-one nursing care, access to respiratory support allowing for the flexibility of management with sedating analgesics, and less distress to the child and family), there are also some important disadvantages. Bed availability in PICU is finite and, if no bed is available on the day of surgery, cancellation and delays can occur. Moreover, the PICU is a high-acuity and -intensity unit, and this may prove challenging to some children and families. Another consideration at our hospital is that parents cannot sleep overnight in the PICU if the patient is intubated, which may distress some families.

A plan is developed and documented in the Electronic Medical Record (EMR) as a Case Conference note and a copy sent to the family and primary care team, summarising the discussion and plan. The decision as to whether the child is safe and a suitable candidate to proceed with surgery is made at the Meeting. Sometimes there is disagreement between the clinical team and the family about whether to proceed with surgery. In these cases, the option of an additional clinical assessment, including the orthopaedic surgeon, the neurodevelopmental paediatrician and other members of the child’s family and care team, is offered. If surgery is ultimately offered, the final decision to choose not to go ahead with the surgery lies with the parents. These examples highlight that the service is not just a preoperative assessment clinic. Decisions are reached from the multi-disciplinary team clinic with surgical expertise, the child and their family at the centre, and the physicians.

A letter is sent to the parents summarising the outcome of the Clinic and Meeting, and the decision. Furthermore, a pre-admission plan is prepared, covering treatment for constipation, the introduction of gabapentin if no contraindications are present, postoperative gastro-oesophageal reflux medication, and a plan as to whether a nasogastric tube will be inserted peri-operatively (File S1).

Three clinical vignettes outlining typical cases and the process used to reach the decision are presented (File S2).

## 6. Discussion

The perioperative pathways we have developed provide a robust framework that includes parental views and hopes, recognising their role as knowledgeable caregivers, to approach this complex decision-making and ensure the best outcomes for children with SNI. The identification of medical morbidity and opportunities to optimise health and anticipatory decisions about the need for post-operative PICU care are important components of this process. The pathway brings together the skill and experience of a range of surgeons and physicians and provides an opportunity for a comprehensive assessment and planning to mitigate the risks inherent to this group. Transparency and honesty in communication is highly valued, particularly when there is uncertainty about the outcome [31,32,33,34,35]. This is the case when counselling for scoliosis surgery, especially when it comes to discussing evidence-based outcomes.

Deciding if an elective surgical intervention is in a child’s best interests can be difficult. The “Best Interests Standard” (BIS) is an ethical, legal, and social principle that has been used to guide decision-making in children’s medical care [36]. The BIS describes a broad cluster of children’s interests, and includes basic needs, emotional development, play and pleasure, to live a long life and to have a relationship with a parent [37]. Multiple approaches to decision-making have been described and all focus on the key principles of the best interests of the child in the context of their family and the minimisation of harm [38].

Decision-making for children with SNI can be complex, and parents and clinicians are often faced with difficult decisions. Shared decision-making [39] in paediatrics is an ideal, in which there is collaboration and flexibility, knowledge and value-related priorities are equal [40]. Nonetheless there are ethical and practical challenges in many clinical situations. Ethical tools, such as the Zone of Parental Discretion (ZPD) [41], have been developed to help clinicians address these ethically complicated cases [38,41]. The ZPD provides a way to explore difficult decisions and uncertainty, and to balance parent authority and children’s best interests. This tool is especially useful when there are disagreements. Parents may have a different view about which interests are more important, and this may create disagreement with the clinical team [42,43]. The use of ethical language to frame the clinical problems encountered has been valuable for the team.

The authority of parents as decision-makers for their child is well-described. Parents are recognised to be best placed to make decisions, as they know their child and will be bear the burden of the (medical) decisions they make, although this authority is not without limits [44]. Parents of children with SNIs have had to make many decisions throughout the lives of their children, often where there is uncertainty about the outcome [45]. Parents expect to be recognised as experts in their own child and, therefore, to warrant an important role in decision-making [42,46,47,48,49]. Parents of children with SNIs are strong advocates for their child [32,46] and emphasise the personhood of their child to the clinical team [31,47]. It is important for parents to feel heard and understood, and for their expertise as knowledgeable caregivers to be recognised. The burden and emotional impact of these complex decisions is also recognised for parents and their children [34].

The Clinic appointment allows for a more detailed discussion about the health needs of the child, and for a deeper exploration of the hopes, concerns and fears a parent has about surgery. Exploring and acknowledging hopes in decision-making is well-described and critical to the process of shared decision making [46,50]. The parental perspective and voice are important to how a decision is made. A study exploring the experience and satisfaction of parents with this process is currently being undertaken.

An additional benefit of the Clinic is that a small proportion of the children considered for orthopaedic interventions are only known to the orthopaedic team and receive their primary paediatric care outside of our hospital. This includes families who have not sought other mainstream paediatric care for their child. The Clinic assessment sometimes brings about the need for a thorough work-up, and multiple interventions prior to surgery. There are benefits for the child and family to meet the broader team and have some familiarity with the other clinical services that will be involved in the child’s care in the perioperative period.

Furthermore, decisions around perioperative care are not always directly in line with previous decisions to not intervene medically. Sometimes, in order to stabilise the child for scoliosis surgery a cascade of medical investigations and interventions are undertaken; for example, a nutritional assessment in a child who is underweight and has an unsafe swallow, commencing enteral feeding or investigation of sleep-disordered breathing, which is longstanding but was previously not explored.

The planning of scoliosis surgery in a child with a clear life-limiting condition and known to the palliative care service can be seen as a confusing active intervention for families. There may also be pre-existing limitations to active interventions or resuscitation orders (although active treatments and involvement of palliative care can co-occur). Note that limitations on resuscitation orders are suspended during the perioperative period. This occurs following careful and explicit discussion with the family and team to ensure that the child can survive the surgical process.

This pathway represents a successful collaboration across multiple craft groups. Clinicians from the Divisions of Medicine, Surgery, Critical Care and Allied Health, alongside research partnerships with MCRI, are involved and meet regularly while providing clinical care. The Meeting has created a space in which the members of the clinical team have developed a greater understanding of the needs, responsibilities, and skills inherent to each other’s roles. Familiarity and respect have evolved and, over time, have created an environment in which open, sometimes vigorous, and respectful discussions occur to support this complex decision-making. This relational capacity has extended beyond the meeting space and has facilitated communication between teams on the ward. The importance of establishing respectful and functional relationships in clinical care is not a new concept [51], but may be one that needs to be given a higher priority when planning complex clinical care, particularly where there is uncertainty about the outcomes. A culture of respect and open communication is recognised to improve patient safety and clinical care, and to increase the meaningfulness and joy of the work of clinicians [52,53]. The collaborative nature of this process has benefits beyond that of decision-making and planning, and has recognised benefits for clinical care, clinical relationships, and staff well-being [51,52].

## 7. Conclusions

The decision to go ahead with scoliosis corrective surgery in children with complex disability and medical comorbidity is a substantive one for both parents and the clinical team. Sometimes significant changes are required before the child is medically ready. The Clinic creates an opportunity to meet each child and family in a setting that is separate to the surgical clinic and allows for an additional opportunity for an exploration of parents’ hopes, wishes, and fears, and to understand the values and beliefs of the family. This important information can then be shared with the broader team at the Meeting and coupled with a detailed assessment of health, potential risks and identified benefits, can be incorporated into both decision-making and planning for perioperative care. Clinical pathways of perioperative care have been developed at our hospital to provide comprehensive support for the care of children with SNI and medical complexity who need orthopaedic surgery. These pathways have created opportunities for supported, collaborative and inclusive decision-making. These pathways provide guidance for optimisation of health prior to surgery and have created improved staff relationships with positive impacts on care.

## Supplementary Materials

The following supporting information can be downloaded at: https://www.mdpi.com/article/10.3390/jcm11226769/s1, Table S1—Clinic Meeting proforma. This figure shows the template used in the Medical Neuromuscular Scoliosis Clinic and the Neuromuscular Scoliosis Multidisciplinary Meeting. File S1—Letter to parents/carer proforma. This figure shows the template used in the letter for the parents. File S2—Clinical vignettes. Reference [54] are cited in the supplementary materials.

## Abbreviations

Best Interests Standard (BIS), Cerebral Palsy (CP), Electronic Medical Record (EMR), Gross Motor Function Classification Scale (GMFCS), Murdoch Children’s Research Institute (MCRI), Pre-Anaesthetic Review Clinic (PARC), Paediatric Intensive Care Unit (PICU), Severe Neurological Impairment (SNI), Zone of Parental Discretion (ZPD).
