# Supplementary material for: Perioperative Care of Children with Severe Neurological Impairment and Neuromuscular Scoliosis—A Practical Pathway to Optimize Peri-Operative Health and Guide Decision Making
Source: J Clin Med. 2022 Nov 16;11(22):6769. doi: 10.3390/jcm11226769 (PMC9696380; doi:10.3390/jcm11226769)
Supplement: Supplementary file 1 [file jcm-11-06769-s001.zip › File S1 Letter to parents Submission.pdf]

File S1 – Letter to parents / carer and primary team proforma. This File shows the template used for the letter for the parents.

Dear Parent of X,

Pre-operative Medical Management Plan
This plan is to start in the two-week period prior to X's orthopaedic surgery. You will have the prescriptions for the medications at home with you so that X can start the medications when you have a date for surgery.

Gabapentin
This will help manage pain and reduce muscle spasm after the surgery. Start this medication in the two weeks prior to surgery.

- *Week one 100 mg orally three times a day orally/PEG/PEJ*
- *Week two 200 mg orally three times a day orally/PEG/PEJ*

This should continue during the admission for the surgery and into the post-operative period and can be weaned 4-6 weeks after surgery.

Anti-reflux medication
Surgery can be stressful and make reflux worse. Fasting and the medications used to manage pain after surgery can also make reflux worse. This is a preventative measure to control reflux. Start this medication in the two weeks prior to surgery.

- *Omeprazole 10-20 mg once a day orally/PEG/PEJ, or*
- *Esomeprazole 10-20 mg once a day orally/PEG/PEJ*

This should continue during the admission for the surgery and can be ceased at time of discharge.

Bowel Care
Constipation is very common after surgery. In the days leading up to the surgery, management of constipation is important

- *Microlax© enema PR one to two days prior to surgery*
- *Increase in lactulose/Movicol©/Osmolax© in the week prior to surgery*

Nasogastric tube
Many children with cerebral palsy/physical disability/Rett's syndrome manage their oral diet well when they are well. After surgery, both pain and medication can make children sleepy, can make swallowing more difficult. It is suggested that a nasogastric is inserted whilst X is asleep during the surgery. It will help ensure usual medications can be given, fluids and feeds can be started, pain medications can also be given, and X will not be dependent on an intravenous drip. If the tube is not needed, it is very easy to remove.

Yours sincerely,
