# Supplementary material for: Perioperative Care of Children with Severe Neurological Impairment and Neuromuscular Scoliosis—A Practical Pathway to Optimize Peri-Operative Health and Guide Decision Making
Source: J Clin Med. 2022 Nov 16;11(22):6769. doi: 10.3390/jcm11226769 (PMC9696380; doi:10.3390/jcm11226769)
Supplement: Supplementary file 1 [file jcm-11-06769-s001.zip › File S2 Vignette.pdf]

Vignette 1

Rose is a 12-year-old girl with bilateral cerebral palsy, mixed motor type, functioning at GMFCS V level, with an uncertain aetiology.

Rose was born at term and had neonatal seizures. Her MRI shows mild ventriculomegaly and Whole Exome Sequencing (WES) has identified a Variant of Unknown Significance (VUS) described as having low clinical relevance.

Rose has poor bulbar function (unsafe swallow), a history of vomiting with secondary aspiration. She had a gastrostomy and fundoplication at 18 months of age. Despite her fundoplication, Rose continues to have intermittent vomiting and haematemesis, and is prone to acute dehydration complicated by hypernatremia – she has had two admissions to the intensive care unit. She has had long term management with a proton pump inhibitor (omeprazole 10 mg *OD*). Rose has daily management for constipation (polyethylene glycol 13.8 g *OD PRN*). She weighs 25 kg. Rose has a complex respiratory history, with multiple respiratory admissions and only one admission to intensive care unit for respiratory symptoms, requiring a short period of non-invasive ventilation. Rose drools anteriorly and has trouble managing her upper airway secretions, with significant pharyngeal pooling. She also has a chronic productive cough (yellow sputum) and has had prolonged courses of antibiotics post-admissions with some improvement in the cough. Rose had polysomnography eight months prior to the Clinic appointment, which did not show obstructive or central apnoea. Rose is on a high dose medication regime (hyoscine hydrobromide transdermal patch - 1.5 mg/72 hours) and enteral glycopyrrolate (40 mcg/kg *qid*), requires frequent suctioning and chest physiotherapy (provided by her mother) throughout the day, and on at least one occasion overnight. Rose has high tone and dystonic posturing of her arms, trunk and her head and neck. Rose is managed with enteral baclofen (25 mg *qid*), gabapentin (400 mg *tds*) and diazepam (2 mg *tds* PRN) with some benefit.

Rose had hip surgery (medial hip release and bilateral proximal femoral osteotomies) two years ago, followed by the removal blade plates 12 months later, and had managed both surgeries well with good post-operative recovery.

Rose has a lumbar scoliosis of 90 degrees (Figure 1) with severe pelvic obliquity. The history obtained in the Clinic was of an ongoing daily productive cough, difficult secretion management, and high suction and chest physiotherapy requirements.

On examination, Rose had nasal congestion, marked drooling, and a persistent open mouth posture. Her chest was clear. The findings were suggestive of suppurative lung disease, secondary to recurrent aspiration and her difficulty in managing her upper airway secretions.

Rose's mother was worried about how quickly her scoliosis had progressed and the secondary impact on her hips. Rose could only spend short periods of time in her wheelchair, and this was starting to limit her ability to attend school – an activity she loves. She felt Rose was increasingly in pain and worried about further respiratory deterioration. She was most worried about Rose's recovery following the surgery. Rose had a chest radiograph done, and routine bloods and a sputum collected for culture following the appointment.

Rose was discussed at the Meeting. Her scoliosis was likely to progress and further impact on her respiratory function, seating, comfort, and participation. Rose was recognised to have respiratory vulnerability, but with potentially modifiable factors. Her chest radiograph (see Figure 2) was suggestive of chronic infection and her sputum culture grew *Moraxella catarrhalis* and *Haemophilus influenzae* (not type B). As part of her pre-surgery assessment, Rose was reviewed by the anaesthetist in the Pre-Anaesthetic Review Clinic (PARC) and by the Intensive Care Physician. Rose was referred to the Saliva Control Clinic, and a recommendation for salivary gland excision and ligation was made, and she was placed on a surgical waiting list

for this procedure. Management to focus on her suppurative lung disease was initiated – she commenced on a four-week course of enteral antibiotics (amoxycillin-clavulanic acid**,** 520 mg-75 mg *bd*). Rose had marked improvement initially, but again deteriorated following cessation of antibiotics. Rose was placed on a prophylactic course of enteral antibiotics (thrice weekly azithromycin, 250 mg). In the lead up to her scoliosis surgery, Rose was admitted for insertion of a long line, and transferred for care to in the Hospital In The Home (HITH) service for a two week course of intravenous antibiotics (ceftriaxone 1.3 g *IV* daily), with home based intensive airway clearance therapy. Her perioperative plan included an increase in her omeprazole and gabapentin doses, and elective admission to the intensive care unit post her surgery.

Rose was admitted for Bipolar posterior spinal corrective surgery[1], and as planned electively admitted to the intensive care unit for respiratory support post-extubation. Rose was transferred to the ward day two post-surgery, having required HiFlow for 48 hours after extubation. On day four post-surgery she developed increasing production of yellow sputum and was commenced on two-week course of enteral antibiotics (trimethoprim-sulfamethoxazole, 100 mg-500 mg, *bd*) and discharged day five postoperatively to HITH for a week of physiotherapy. Rose is now 11 months post-spinal corrective surgery.

[1] Lofti, M.; Gaume, M.; Khouri, N.; Johnson, M.; Topouchian, V.; Glorion, C. Minimally Invasive Surgery for Neuromuscular Scoliosis: Results and Complications in a Series of One Hundred Patients. *Spine* **2018**, *43*, E968-E975, doi: 10.1097/BRS.0000000000002588.

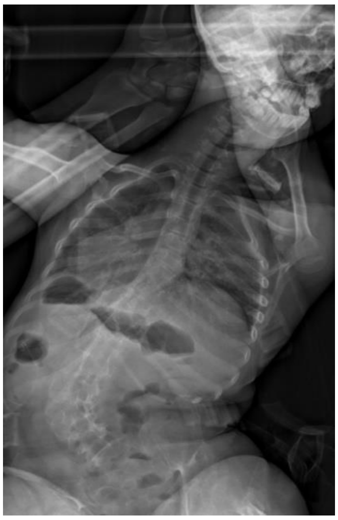

Figure 1
Severe levoconvex scoliosis of the lumbar spine centred in the mid lumbar region

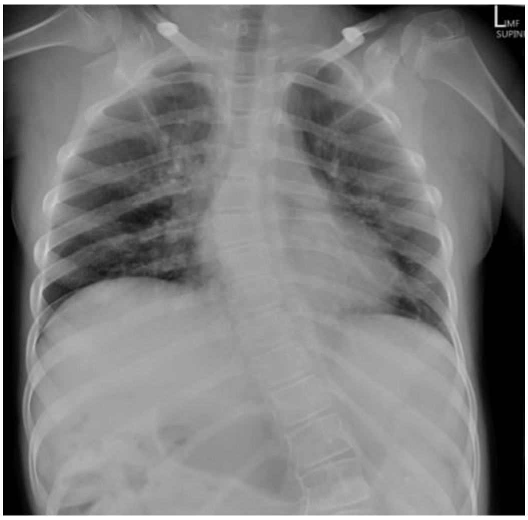

Figure 2
Prominent perihilar peri-bronchial thickening is demonstrated bilaterally. There is persistent left lower lobe patchy opacity/atelectasis. Ill-defined opacity is seen within the right upper lobe associated with bronchial dilatation suspicious for underlying bronchiectasis. The lung volumes are maintained and there is no drainable pleural fluid collection or pneumothorax.

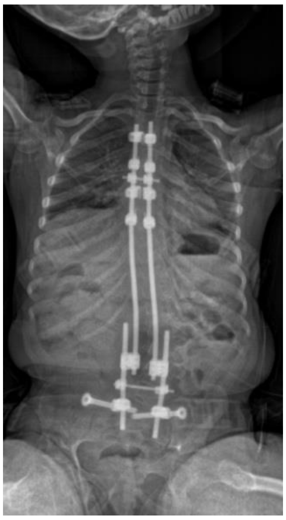

Figure 3
Spinal correction post Bipolar instrumentation

Vignette 2

Emir is a ten-year-old boy with a moderately severe intellectual disability and a communication disability. Emir has a motor disability – he is non-ambulant, able to do standing transfers, and functions at a GMFCS IV-equivalent level. Emir has high sensory needs and behaviours, including lip-smacking, grunting, and sniffing with secondary aerophagia. Emir had a microarray done when he was three years old, and this was uninformative. Emir takes a modified oral diet and there is no recent history suggestive of bulbar dysfunction – he weighs 22 kg. He has a history of mild constipation. He takes no medications on a regular basis. As his scoliosis has progressed, his ability to sit well in his wheelchair has changed and his mother describes he now coughs as he eats. His mother did not report any concerns that Emir was experiencing pain. Emir has always been well – his last hospital admission was at three years of age with a respiratory illness. Emir sees a paediatrician every 12-18 months and the family are supported by their general practitioner. Emir attends school in a special setting, and the staff have reported some events at school, which they are concerned may be seizures. The progression of his scoliosis and the unusual events at school prompted his mother to arrange an appointment with his paediatrician, who made a referral for an orthopaedic review and outpatient EEG.

Emir has a thoracic scoliosis of 70 degrees (Figure 1) with minimal pelvic obliquity. The history taken at the Clinic noted that Emir was a mouth breather and would typically sleep 13-14 hours each night - he needed to be woken to go to school and was sometimes difficult to rouse. There was no other history suggestive of obstructive sleep apnoea. The episodes at school were described as periods of staring and hyperventilation, from which he could not be distracted. The events were short (1-2 minutes), occasionally he would have a brief sleep after the event. They had increased in frequency over recent months. He was also reported to be more tired at school.

On examination Emir was noted to have some unusual facial features – mild synophrys, a beaked nose, a large mouth, and widely spaced teeth. Emir had almost continuous movements of his lips, mouth, and hands, with notable grunting and air swallowing. His respiratory examination was unremarkable.

Emir's mother described that Emir had always been a well boy and she was concerned about the changes in his spine. She wanted him to be able to sit better and continue to eat as well as he had always done. She was also worried that his spine would get worse, but was worried about surgery, and the risk of having an anaesthetic.

Emir's case was discussed at the Meeting. Emir's scoliosis was likely to continue to progress and surgery was indicated. It was acknowledged that he had an unremarkable medical history, however, there were concerns about his risk of obstructive sleep apnoea and seizures. In addition to completion of a panel of nutritional bloods, overnight oximetry was planned. His pre-operative plan additionally included the recommendation to start a proton pump inhibitor and aperients leading up to the surgery. It was also recommended that Emir have a nasogastric tube to support him in the post-operative period. A decision regarding the need for elective admission to paediatric intensive care would be made once further investigations had been completed

Emir's overnight oximetry revealed frequent clusters of desaturation, and an elective admission was arranged. On admission his capillary blood gases were pH 7.33, pCO2 43 mmHg, bicarbonate 22 mmol/L. Emir's sleep study demonstrated mixed sleep disordered breathing, with a moderately severe obstructive component, prolonged central apnoeas (max 28.5 secs) and associated mild hypoxaemia. He was reviewed by otolaryngology who noted adenoidal enlargement, with obstruction of the choanae, marked nasal secretions and no other abnormalities on examination. Treatment with mometasone and xylometazoline nasal sprays were recommended with follow up outpatient review to consider role of adenoidectomy.

During this admission Emir was commenced on non-invasive ventilation (BiPAP, pressures 10/4, RR 18/min), which he tolerated well.

Emir had an EEG – it did not capture any of the described events, and he is awaiting ambulatory EEG monitoring. During the admission, Emir was reviewed by Genetics, and a Whole Exome Sequence (WES) study was completed. The results of the WES showed Emir has Pitt-Hopkins Syndrome. He is awaiting scoliosis corrective surgery.

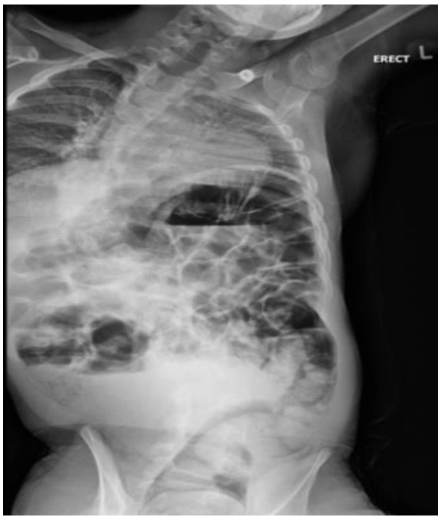

Figure 1
Thoracic scoliotic curve convex to the right

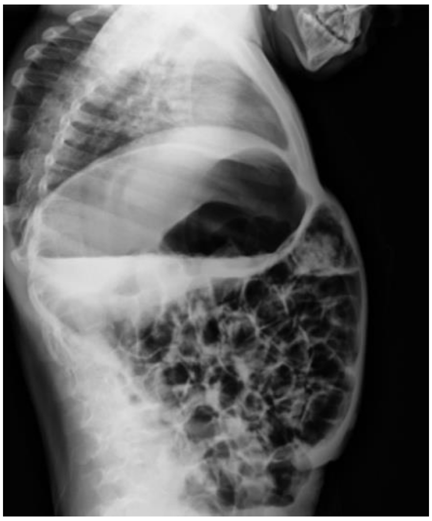

Figure 2
Marked gaseous distension of the stomach with multiple gas filled loops of bowel in the upper abdomen.

Vignette 3

Noah is a 16-year-old adolescent with bilateral cerebral palsy, mixed motor type, functioning at a GMFCS V level in the context of delivery at 27 weeks gestation. Noah has problematic dystonia and a very strong atonic neck reflex. Noah has chronic pain, with likely multiple sources. The management of both his pain and dyskinesia has been challenging as he been sensitive to a range of medications - baclofen, benzodiazepines and opiates cause marked sedation, with an impact on his respiratory function. He is currently taking tetrabenazine (12.5 *mane*, 18.75 mg *nocte*), gabapentin (600 mg *tds*) and both ibuprofen and paracetamol daily. Noah had bilateral femoral osteotomies (done at his local hospital) five years ago and he required admission to the intensive care unit after that surgery because of respiratory deterioration and difficult to manage pain. His right hip is now dislocated (Figure 1). Noah had a gastrostomy at two years of age; he is now gastro-jejunally fed secondary to recurrent vomiting and haematemesis, aspiration and poor feed tolerance. Noah needs to be fed slowly as he often feels uncomfortable and readily gags. Noah had an episode of pancreatitis 18 months ago. Noah weighs 31 kg. Noah has a significant respiratory history – he has severe obstructive sleep apnoea. Noah's symptoms did not improve following adenotonsillectomy and he has been on non-invasive ventilation (NIV) and supplemental oxygen at night for about two years. Noah does not tolerate this very well and must be carefully positioned each night. He has had three admissions to the intensive care unit for respiratory illnesses in the last two years, and on one occasion required intubation. Noah is known to the local palliative care team who have initiated discussions with the family about advanced care. Both his mother and father had described that intensive care was a "*hard place for Noah to be*" and would not want him to be intubated again or to have "*chest compressions*".

Noah has a lumbar scoliosis of 100 degrees. (Figure 2), with severe pelvic obliquity. The history obtained in the Clinic suggested that Noah had daily pain and was often tearful and distressed. Noah was noted to have marked dyskinesia, with almost continuous movements and posturing in his wheelchair - he was very agitated and uncomfortable.

Noah's parents voiced their concern about how rapidly his spine had changed. They were both worried about "*how twisted*" Noah had become and about the impact this would have "*on all his organs*". They noted it was hard for him to spend time in his wheelchair and he had been unable to get to school often in the last 12 months. His parents described they hoped the surgery would substantially reduce his pain and allow him to sit in his chair and go to school.

Noah's case was discussed at the Meeting and several concerns were raised – his respiratory fragility, his difficult to manage dyskinesia, and his sensitivity to medications. His parents had indicated they wanted Noah to have surgery, but there was an advanced care plan in place. There was concern about the risk of a prolonged period of intubation, and a very prolonged inpatient stay. It was also recognised that whilst his scoliosis was a contributor to his pain, he had multiple potential sources of pain – including his hip – and that surgery would not resolve and could potentially worsen his pain.

A further appointment was arranged with Noah's parents and his local care team – his paediatrician, respiratory physician, physiotherapist, and staff from the palliative care team. We again explored his parents' concerns and expectations of the surgery – his parents both said they just assumed the "*surgery would happen*" and would resolve Noah's pain. Noah's medical fragility and likely prolonged and complex recovery was described; however, our greater concern was that this surgery would not reduce Noah's pain and could potentially make him worse. It was very challenging for the family to consider the option of no surgical intervention, but they were focussed on improving his quality of life. This facilitated a discussion about how this might be achieved for Noah with support of the team around him. It was agreed that proceeding with scoliosis corrective surgery was not in Noah's best interest at this time.

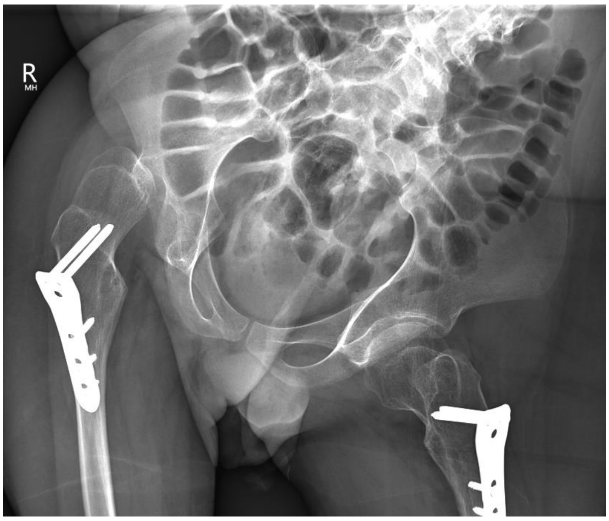

Figure 1
Pelvic x-ray demonstrating previous surgery to Noah’s hips, marked pelvic obliquity, and evidence of a dislocated right hip

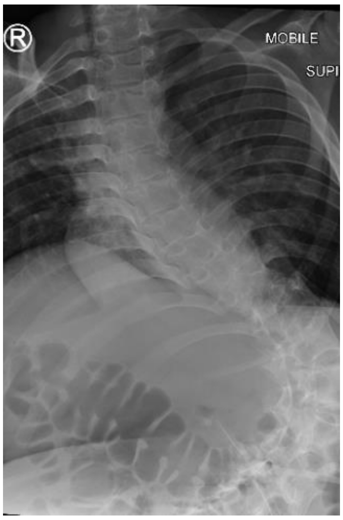

Figure 2
Radiology of spine demonstrating extent of Noah’s lumbar scoliosis
