# Supplementary material for: Perioperative Care of Children with Severe Neurological Impairment and Neuromuscular Scoliosis—A Practical Pathway to Optimize Peri-Operative Health and Guide Decision Making
Source: J Clin Med. 2022 Nov 16;11(22):6769. doi: 10.3390/jcm11226769 (PMC9696380; doi:10.3390/jcm11226769)
Supplement: Supplementary file 1 [file jcm-11-06769-s001.zip › Table S1 - Meeting proforma Submission.pdf]

Figure S1 – Meeting proforma. This shows the template used in the Neuromuscular Scoliosis Multidisciplinary Meeting (with details from the Clinic below).

Case Conference
Neuromuscular Scoliosis Combined Clinical Meeting

Date	
**Participants**	
Names of attendees	
**Has consent been obtained from the patient/family?** YES	
**Outcome of Medical Scoliosis Clinic**	
Further investigations required	
Recommendation – suitable vs not suitable for surgery	
Nutritional bloods	
Elective PICU admission post-op recommended	
**Discussion Notes and Plan**	
**Medications changes since clinic**	
**Recent bloods** *date*	
**Ferritin** ug/L	
**Hb**	g/L
**PCO2**	mmHg
**Additional pre-operative investigations/assessments**	
**Pre-operative PICU review** – Yes / No	
**Pre-operative plan**	
**Nasogastric tube intra-operatively** – Yes / No	
**Elective PICU admission post-op recommended** – Yes / No	
**Radiology reviewed**	
**Duration of discussion**	

Information proforma from the Clinic

**Name**	
**MRN**	
Date of Birth	
Date reviewed in Clinic	
Attended Clinic with	
Paediatrician	
Scoliosis surgeon	
Scoliosis Curve (Radiology date)	
Orthopaedic Surgical Plan	

Summary of Medical Review

**Diagnosis**<br>MRI/Genetic	
Problem list	
Current medications	
Current weight	
**COVID**	Infection: Yes / No<br>Date:<br>Immunizations status:<br>Child/adolescent:<br>Family/Carers:
**Influenza**	Immunizations status:
**Respiratory Status**	**Baseline**<br>Chronic Lung Disease – prematurity:<br>Suppurative lung disease:<br>Chronic cough:<br>Aspiration: Primary/Secondary<br>Vomit:<br>Reflux:<br>Asthma:<br><br>**Sleep history**<br>Snoring:<br>Apnoea:<br>OSA:<br>Hypoventilation:<br><br>CXR:<br>Chest CT scan:<br>Sputum culture:<br>VFSS:<br>Oximetry:<br>Sleep Study:<br><br>**Function**<br>Cough:<br>Strength<br>Effective<br><br>Lung function tests:<br>Secretions/Drooling: Yes / No

	**Current Management**<br>Home chest physiotherapy regime:<br>Home suction: Yes / No<br>Home oxygen: Yes / No<br>Home NPA: Yes / No<br>Home APAP/CPAP/BiPAP/NONE:<br><br>Respiratory illnesses last 12 months:<br>Number of respiratory admissions last 12 months:<br>Typical Mx for respiratory illness/duration/recovery:
**Previous admission to PICU and management**	Yes / No<br>HiFlow: Yes / No<br>CPAP: Yes / No<br>BiPAP: Yes / No<br>Intubation: Yes / No<br>Number of admissions to PICU<br>Elective admission post-surgery: Yes / No
**Response to previous Anaesthetics/Surgery**	
**Mobility and function**	GMFCS level<br>GMFCS-equivalent level
**Tone and Movement Ds**	Spasticity: Yes / No<br>Dystonia: Yes / No<br>Management:<br>ITB: Yes / No<br>DBS: Yes / No
**Pain**	Signs of Pain:<br>Mx of pain:
**Epilepsy/Seizures**	Type, frequency:<br>Management:<br>Ketogenic diet: Yes / No
**Communication and Cognition**	Communication:<br>School:
**Mental health, behaviour, sensory issues**	
**Nutrition**	Nutritional Status:<br>Recent weight gain/loss:<br><br>Feeding:<br>Feed regime:<br><br>Vomiting: Yes / No<br>Reflux: Yes / No<br>Fundoplication: Yes / No<br>Constipation: Yes / No
**Bone health**	History of insufficiency fracture: Yes / No<br>DEXA: Yes / No<br>Treatment - bisphosphonates: Yes / No
**Pubertal Status**	Pre/post-pubertal
**Skin Health**<br>**Wound healing**<br>**History of pressure sores**	

**Recent Investigations**	**Date completed**<br>Haemoglobin:<br>Ferritin:<br>CRP:<br>Vitamin D:<br>CAB:<br>Radiology:<br>Oximetry:<br>Sleep Study:

Family/Guardian view

**Family/child/adolescent goal for surgery** (anticipated outcomes/benefits)	
**Family/child/adolescent concerns/ thoughts about surgery**	

Results, Actions and Recommendations

**Additional investigations and information required**	Request Medical records<br>Additional pre-operative investigations/assessments
**Recommendations pre surgery**	Pre-operative admission: Yes / No<br>Pre-operative plan:<br>Nasogastric tube intra-operatively: Yes / No<br>Elective PICU admission post-op recommended: Yes / No
